# The human PRD-like homeobox gene *LEUTX* has a central role in embryo genome activation

**DOI:** 10.1242/dev.134510

**Published:** 2016-10-01

**Authors:** Eeva-Mari Jouhilahti, Elo Madissoon, Liselotte Vesterlund, Virpi Töhönen, Kaarel Krjutškov, Alvaro Plaza Reyes, Sophie Petropoulos, Robert Månsson, Sten Linnarsson, Thomas Bürglin, Fredrik Lanner, Outi Hovatta, Shintaro Katayama, Juha Kere

**Affiliations:** 1 Department of Biosciences and Nutrition, Karolinska Institutet, Novum, Huddinge 141 83, Sweden; 2 Competence Centre on Health Technologies, Tartu 50410, Estonia; 3 Department of Clinical Science, Intervention and Technology, Karolinska Institutet, Karolinska University Hospital Huddinge, Stockholm 141 86, Sweden; 4 Center for Hematology and Regenerative Medicine Huddinge, Karolinska Institute, Stockholm 14183, Sweden; 5 Department of Medical Biochemistry and Biophysics, Karolinska Institutet, Stockholm 171 77, Sweden; 6 Department of Biomedicine, University of Basel, Basel 4058, Switzerland; 7 Science for Life Laboratory, Tomtebodavägen 23 A, Solna 171 21, Sweden; 8 Molecular Neurology Research Program, University of Helsinki and Folkhälsan Institute of Genetics, Biomedicum 1, Haartmaninkatu 8, Helsinki 00290, Finland

**Keywords:** Embryo genome activation, Homeobox gene, Preimplantation development, Embryonic stem cells

## Abstract

Leucine twenty homeobox (*LEUTX*) is a paired (PRD)-like homeobox gene that is expressed almost exclusively in human embryos during preimplantation development. We previously identified a novel transcription start site for the predicted human *LEUTX* gene based on the transcriptional analysis of human preimplantation embryos. The novel variant encodes a protein with a complete homeodomain. Here, we provide a detailed description of the molecular cloning of the complete homeodomain-containing *LEUTX*. Using a human embryonic stem cell overexpression model we show that the complete homeodomain isoform is functional and sufficient to activate the transcription of a large proportion of the genes that are upregulated in human embryo genome activation (EGA), whereas the previously predicted partial homeodomain isoform is largely inactive. Another PRD-like transcription factor, *DPRX*, is then upregulated as a powerful repressor of transcription. We propose a two-stage model of human EGA in which *LEUTX* acts as a transcriptional activator at the 4-cell stage, and *DPRX* as a balancing repressor at the 8-cell stage. We conclude that *LEUTX* is a candidate regulator of human EGA.

## Abstract (precis)

**Highlighted Article:** Analysis of the expression pattern and targets of the human transcription factor LEUTX suggests a two-stage model for embryonic genome activation, also involving DRPX.

## INTRODUCTION

Homeobox genes encode homeodomain-containing transcription factors, which often regulate developmental processes and cellular differentiation (Holland, 2013; Monteiro et al., 2006; Mallo and Alonso, 2013; Seifert et al., 2015). An analysis of human embryo genome activation (EGA), focusing on transcription start sites (TSSs) in oocytes, zygotes and isolated single blastomeres from 4-cell and 8-cell embryos (Töhönen et al., 2015), revealed the expression of many paired (PRD)-like homeobox genes. One of the previously unannotated TSSs implicated in human EGA marks the PRD-like homeobox gene *LEUTX*.

The *LEUTX* gene is characterized by a highly conserved PRD class homeodomain, except for a leucine at position 20, and a lack of paired domain, similar to the other PRD-like transcription factors (Galliot et al., 1999; Bürglin, 2011). Evolutionarily, neither Leutx nor three other PRD-like gene families (Dprx, Argfx and Tprx) are present in invertebrates (Holland, 2013). Closely related sequences for human *LEUTX* have been detected in other primates and highly divergent orthologs are present in several other placental mammals (Zhong and Holland, 2011). *LEUTX* is deduced to have arisen by tandem duplication and divergence from the Otx family gene *CRX* during the early radiation of placental mammals, and it has been subsequently lost from rodents (Holland, 2013; Zhong and Holland, 2011).

The RefSeq model for *LEUTX* (NM_001143832.1) was predicted *in silico* from human genomic sequence (Holland et al., 2007). The annotation was based on computationally predicted mRNA (XM_001129035.1; LOC342900) supported by partial 5′ cDNA sequence and predicted stop codon location. One partial cDNA clone of human *LEUTX* was isolated from a placenta cDNA library in 1995 (IMAGE clone ID: 150840) and the existence of *LEUTX* mRNA was further confirmed in 2005 by PCR from pooled mRNA sources (clone ID: MGC10SS.1.1.L1.1.E01).

Our recent study on human preimplantation development indicated expression from a novel TSS in the first intron of the RefSeq *LEUTX* sequence (Töhönen et al., 2015). Here, we provide a full description of the molecular cloning of the embryonically expressed human *LEUTX* encoding a complete homeodomain (hereafter *LEUTX.*n). We show experimental evidence of *LEUTX* expression in human embryonic stem cells (hESCs) and 8-cell stage embryos and a survey of publicly available expression profiles. By overexpression of *LEUTX.*n in hESCs we identify its target genes. We also suggest that the LEUTX.n isoform can act via the 36 bp DNA motif that is found enriched among upregulated genes during human EGA (Töhönen et al., 2015). We observed that ∼25% of the genes upregulated in 8-cell embryos represent experimentally validated target genes for the *LEUTX*.n isoform. *DPRX* acts as a suppressor of a large number of overlapping target genes. Our findings suggest that human *LEUTX* might act as a main regulator of EGA.

## RESULTS

### Cloning of a complete homeodomain *LEUTX* isoform from human preimplantation embryos

Our TSS-focused RNA sequencing data on human preimplantation development (Töhönen et al., 2015) suggested the expression of a variant of *LEUTX* from a previously unannotated TSS within the first intron of the predicted gene, and we verified the full sequence by cloning from a single 8-cell stage embryo library. In the current study, we validate the cloning using cDNA libraries from three whole 8-cell embryos.

**Fig. 1.**
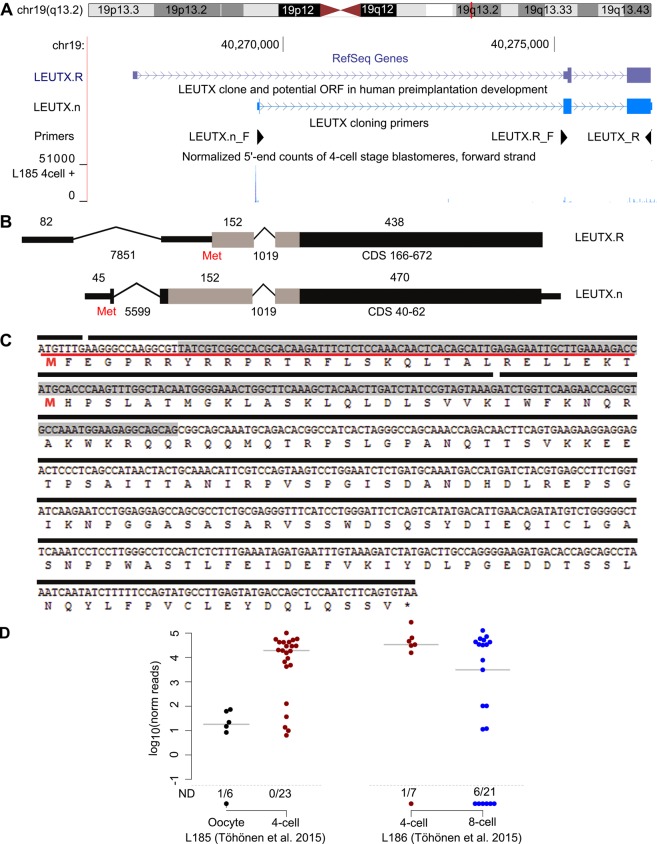
**cDNA cloning and expression of human *LEUTX* in preimplantation embryos.** (A) Schematic representation of the *LEUTX* chromosomal region, *LEUTX* isoforms and primers used in cloning. The blue histograms at the bottom visualize the gene expression in single human 4-cell blastomeres (Töhönen et al., 2015). The detected reads cluster in the middle of the first intron of *LEUTX.*R. (B) Intron-exon structures of *LEUTX.*R and *LEUTX.*n. Boxes represent exons (drawn to scale), with homeobox in gray; thick lines represent 5′ and 3′ UTRs; thin lines represent introns. Nucleotide length is given above exons and below introns. The size of the coding DNA sequence (CDS) is indicated. Red ‘Met’ marks the first methionine codon in transcripts. (C) Experimentally deduced *LEUTX.*n cDNA sequence and exon structure of the predicted ORF. The exons are marked by black bars with gray shading indicating the homeobox sequence. Red bar marks the additional coding sequence in *LEUTX.*n. The first methionine (M) of each is highlighted in red. Amino acid sequence is given beneath. (D) Single-cell gene expression in two sequencing libraries including (left) oocytes (*n*=6) and 4-cell stage blastomeres (*n*=23) and (right) 4-cell (*n*=7) and 8-cell (*n*=21) stage blastomeres as Log_10_ transformed expression values. ND, not detected. Bars indicate median values.

In order to clone the putative new *LEUTX* transcript, we designed a forward primer at the observed TSS at position chr19:40269483 (GRCh37/hg19) and the reverse primer at the predicted 3′ UTR of *LEUTX* (Fig. 1A). PCR yielded a single amplicon (Fig. S1), which was sequenced and found to include an unannotated 5′ exon spliced into exons 2 and 3 of *LEUTX*.

An analysis of the cDNA sequence revealed an open reading frame (ORF) with a complete homeodomain in the novel *LEUTX* isoform (*LEUTX.*n), whereas the RefSeq *LEUTX* (hereafter *LEUTX*.R) would encode a protein with a partial homeodomain (Fig. 1B). The new first exon contains a translation initiation codon (ATG), adding 30 amino acids upstream of the previously known start codon. The experimentally confirmed new cDNA and the translated amino acid sequence are shown in Fig. 1C. The single-cell resolution TSS expression data (Töhönen et al., 2015) indicated a peak in *LEUTX*.n expression in 4-cell stage blastomeres and continued expression at the 8-cell stage (Fig. 1D).

### *LEUTX* is not conserved in mouse

To explore the conservation of *LEUTX* and its function in mouse, we performed a blastp search against the latest NCBI non-redundant database using our new human LEUTX amino acid sequence as a query. The most similar sequence was XP_006544220.2 (‘PREDICTED: leucine-twenty homeobox isoform X1'; E=6×10^–15^; 36% identity in the 60-190 amino acid range of human LEUTX). By UCSC Blat aligner, the corresponding mRNA sequence XM_006544157.2 aligned to chr7:28243022-28243753 of mouse reference genome sequence (mm10) without exon splicing. The ORF begins with ten copies of MPVS(E/G)(A/S)(S/L)(S/I)N(Q/P)A repeats, but it lacks the cognate homeodomain. With a lower amino acid sequence identity, other similar sequences were NP_031796.1 and NP_001106801.1 (cone-rod homeobox protein isoform 1 and 2; E=2×10^–9^ and 3×10^–9^, respectively; 32% identity in the 4-166 amino acid range of human LEUTX), but these were more similar to mouse *Otx1* and *Otx2*; therefore, the alternative name of this cone-rod homeobox protein is Otx3 (also known as Crx) (Chen et al., 1997). There were no alignments of human LEUTX amino acid sequences against mm10 by UCSC Blat aligner with translation. Thus, we confirm the earlier results of Zhong and Holland (2011) that mouse lacks the orthologous gene for human *LEUTX*.

### High-level expression of *LEUTX* is restricted to early embryos

**Fig. 2.**
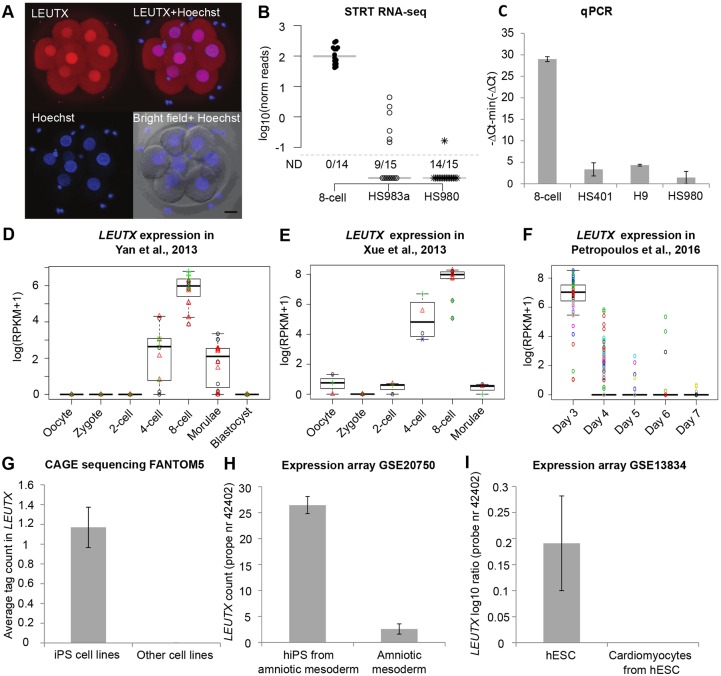
***LEUTX* expression in human preimplantation embryos and pluripotent stem cells.** (A) Indirect immunolabeling in nuclei (blue) indicates LEUTX expression (red) in the human 8-cell embryo (*n*=3). Scale bar: 20 µm. (B) STRT sequencing of single cells from human 8-cell blastomeres (*n*=14) and two hESC lines (HS983a and HS980, *n*=15 for each). The TSS-specific reads from *LEUTX.*n show low or undetectable expression in hESCs, but detectable expression in 8-cell blastomeres. ND, not detected. (C) qPCR validation in three hESC lines and an independent 8-cell embryo library. Expression was detected in all studied cell lines (*n*=3), but only in one replicate for HS980. (D-F) *LEUTX* expression in Yan et al. (2013), Xue et al. (2013) and Petropoulos et al. (2016) data, all supporting *LEUTX* expression in 4- and 8-cell embryos and downregulation in the morula/blastocyst. (F) Day 3 to day 7 refer to embryos from the 8-cell stage to late blastocyst. (G) FANTOM5 data for *LEUTX* (*n*=1829). Only six iPSC samples contained tag clusters in the *LEUTX* region. The average expression refers to the FANTOM5 CAGE phase 1 and 2 samples in which expression was detected. (H) Human somatic fibroblasts from amniotic mesoderm (*n*=3) were used to generate human iPSCs (hiPS, *n*=15) (GEO dataset GSE20750, probe 42402). *LEUTX* shows higher expression in human iPSCs. (I) hESCs (*n*=4) were differentiated into cardiomyocytes (*n*=4) and analyzed by RNA expression array (GEO dataset GSE13834, probe 42402). *LEUTX* was expressed in hESCs, but not in differentiated cardiomyocytes. *LEUTX* is detected by Agilent 014850 Whole Human Genome Microarray 4x44K G4112F (H,I). Error bars indicate mean±s.e.m.; all comparisons for G-I are statistically significant (Student's *t*-test, *P*=0.05).

In order to confirm the expression level of LEUTX protein in human preimplantation embryos, we studied three human 8-cell embryos by immunostaining. The results showed the presence of LEUTX in all apparently normal blastomeres, with prominent nuclear staining (Fig. 2A). The specificity of the staining was confirmed by overexpressing GFP-conjugated LEUTX.n in hESCs and human embryonic kidney cells (HEK-293), and labeling the cells with the same LEUTX antibody with and without competing peptide (Fig. S2).

To study *LEUTX* expression in pluripotent cells, we subjected single cells from two different hESC lines and 8-cell embryos to single-cell tagged reverse transcription (STRT) sequencing (Islam et al., 2012). STRT is an RNA-sequencing method that can be applied to single cells or low amounts of RNA. The method detects the very 5′-end of the poly(A)^+^ transcripts and allows simultaneous analysis of 48 or 96 multiplexed samples. Our sequencing library comprised 15 cells from the inner cell mass-derived hESC line HS980, 15 cells from a single 8-cell blastomere-derived cell line HS983a (Rodin et al., 2014) and 14 individual 8-cell blastomeres from two different embryos. *LEUTX* was detectable in 6/15 HS983a cells and one of the HS980 cells (Fig. 2B) and in all 8-cell blastomeres. Using qPCR, we detected low-level *LEUTX* expression in the hESC lines HS980, HS401 and H9 (Fig. 2C), whereas the expression level of *LEUTX* in an 8-cell embryo was significantly higher.

To further confirm *LEUTX* expression in human preimplantation embryos, we extracted *LEUTX* expression patterns from three previously published RNA-seq datasets (Yan et al., 2013; Xue et al., 2013; Petropoulos et al., 2016). These results are in agreement with our data showing that *LEUTX* is expressed at the 4- and 8-cell stages and is reduced again in the morula and at the latest blastocyst stage (Fig. 2D-F). Owing to methodological differences, the highest peak is detected either at the 4-cell or 8-cell stage (Fig. S3).

The expression of the *LEUTX.*n isoform across different tissues and cell culture models was assessed in the functional annotation of the mammalian genome (FANTOM5) database (Forrest et al., 2014; Lizio et al., 2015). FANTOM5 facilitates systematic investigation of the gene expression profiles in all human cell types, including TSS data from 1829 biological samples. The FANTOM5 data showed a total of seven normalized reads aligned to the *LEUTX.*n promoter and no reads corresponding to the RefSeq TSS (Fig. S4). All the reads were found in six human induced pluripotent stem cell (iPSC) line-derived samples, with an average of 1.17 reads per sample (Fig. 2G). The number of reads for *LEUTX* was too low to be recognized as a TSS in the FANTOM5 database. In comparison, the FANTOM5 database shows more than 10,000 normalized reads for *OTX2*, a previously characterized PRD-like gene. FANTOM5 data are thus consistent with our data for both the TSS location and barely detectable expression in iPSCs only.

The expression of *LEUTX* in pluripotent stem cells was assessed further in two GEO datasets, GSE20750 (Saito et al., 2011) and GSE13834 (Cao et al., 2008), representing iPSC derivation from human amniotic mesoderm fibroblasts and hESC differentiation into cardiomyocytes, respectively. Both datasets showed higher expression of *LEUTX* (third exon) in iPSCs or hESCs compared with other samples as assessed by expression microarray (Fig. 2H,I). The data cannot distinguish between the two TSSs.

Very low expression in placenta, but not in other tissues, has also been shown by others using RT-PCR (Chinen et al., 2014) and RNA-seq (Fig. S5A) (Metsalu et al., 2014). This is in concordance with our failure to detect *LEUTX* in different human tissues by western blot (Fig. S6). The observation is strengthened by the fact that *LEUTX* is not detected at the RNA or protein level in any tissue in the Human Protein Atlas (Uhlén et al., 2015). However, Yao et al. (2014) showed that *LEUTX* is upregulated following *DUX4* overexpression in human muscle cells and is expressed in muscle biopsies from facioscapulohumeral dystrophy (FSHD) patients. Interestingly, they also detected the expression of the novel first exon of *LEUTX*.

We then assessed *LEUTX* expression in the publicly available data on 675 human cancer cell lines (Klijn et al., 2015). The data showed *LEUTX* expression levels in three cell lines (KMS.11, KLE and SU.86.86) at very low levels (Fig. S5B). The highest RPKM value (∼20) was detected in SU.86.86, which expressed a suggested fusion transcript of *PAK4* and *LEUTX*. In comparison, the housekeeping gene *GAPDH* showed RPKM values >2000 in all samples. To conclude, both our own data and publicly available datasets suggested that high-level *LEUTX* expression is restricted to early embryos. The databases investigated in this study are listed in Table S1.

### The *LEUTX.*n isoform activates genes in hESCs

**Fig. 3.**
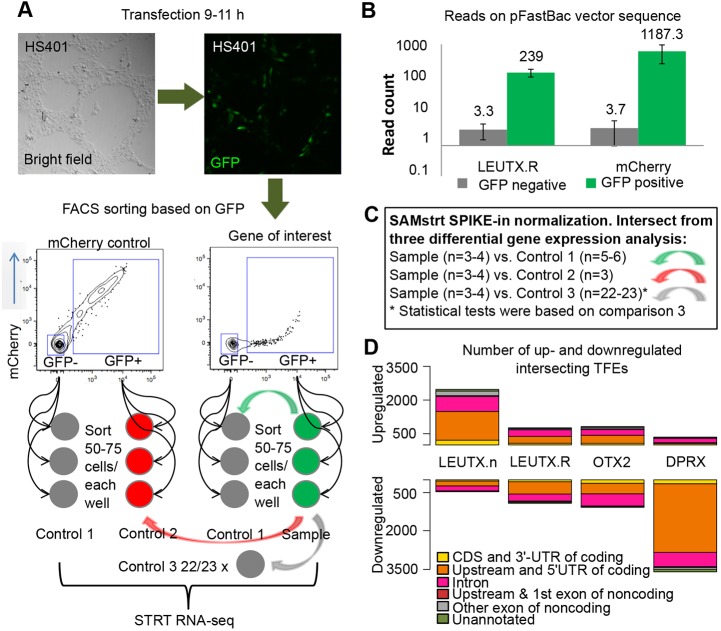
**Experimental set-up for target gene determination.** (A) Outline of the hESC overexpression experiment. *LEUTX*.n, *LEUTX.*R, *OTX2*, *DPRX* and mCherry (control) were overexpressed in hESC line HS401 for 9-11 h using a modified pFastBac vector containing GFP. Then, 50 or 75 fluorescent and non-fluorescent cells were FACS sorted into lysis buffer and used for library preparation. (B) Read alignment on pFastBac vector backbone from *LEUTX*.R and mCherry samples performed after sequencing, quality control and removal of barcodes. GFP-negative cells have few or no reads originating from the vector. Error bars indicate mean±s.e.m. (C) Differential TFE expression analysis and normalization was performed with the SAMstrt package in R using three different sets of controls: (1) GFP-negative samples from both mCherry and the gene of interest (*n*=5 or 6, green arrow); (2) GFP-positive samples from mCherry control (*n*=3, red arrow); and (3) GFP-negative wells from the whole library (*n*=22 or *n*=23 for libraries 1 and 2, respectively, gray arrow). Finally, the gene list from comparison 3 was reduced taking the intersection of all three comparisons (*P*<0.1 for each). Statistical values given in Table 1 and Table S1 are based on comparison 3. The comparisons are marked accordingly with colored arrows in A. (D) The intersecting target genes were annotated to genomic regions.

In order to study the transcription factor profile and target genes of *LEUTX*, we overexpressed both the *LEUTX.*n and *LEUTX.*R isoforms in hESCs. The *LEUTX.*R vector was engineered from our *LEUTX*.n clone to correspond to the RefSeq prediction. The experiment is outlined in Fig. 3A. The genes were cloned into a modified bicistronic pFastBac vector co-expressing eGFP marker, transfected into the hESC line HS401 and sorted in three to four replicates by fluorescence-activated cell sorting (FACS) based on the GFP expression originating from the same vector as the overexpressed gene of interest (Fig. S7). Sorted cells were analyzed using STRT RNA-seq (Islam et al., 2011; Krjutškov et al., 2016). The presence of sequence reads aligning to the modified pFastBac vector backbone sequence in GFP-positive cells, but not GFP-negative cells, confirmed the successful transfection (Fig. 3B). As reference genes for comparisons, we used the previously characterized human PRD-like homeobox gene *OTX2* and the PRD-like homeobox gene *DPRX*, which was significantly upregulated at the 4-cell to 8-cell transition (Töhönen et al., 2015) (Fig. S8). The *DPRX* expression pattern in early human development was confirmed using three independent RNA-seq datasets (Yan et al., 2013; Xue et al., 2013; Petropoulos et al., 2016).

**Table 1.**
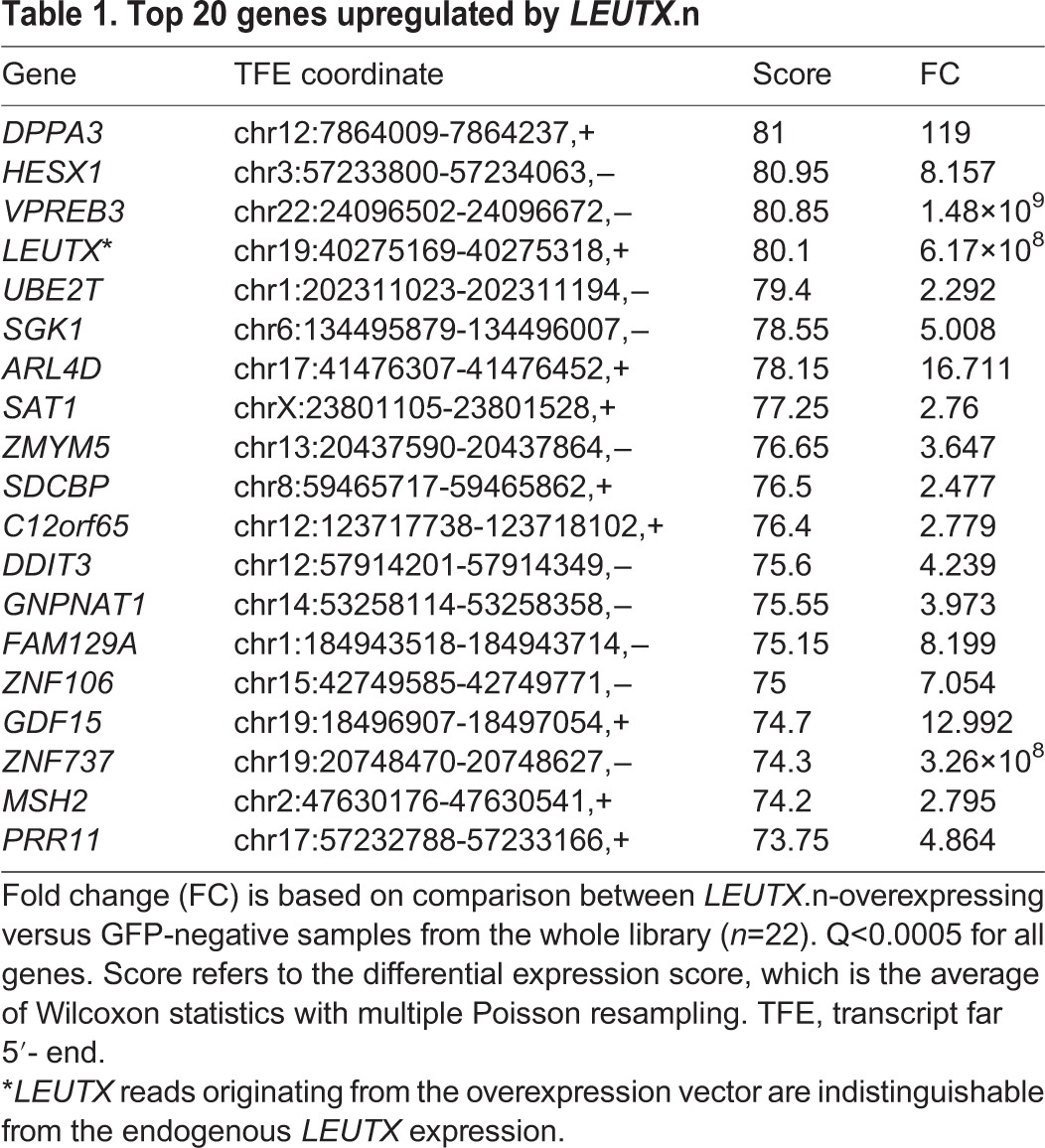
**Top 20 genes upregulated by *LEUTX*.n**

We defined the transcript far 5′-ends (TFEs) for each sample (supplementary Materials and Methods). The differential expression between samples was quantified using SAMstrt, which is a statistical test utilizing spike-in normalization for differential expression sequenced in transcriptomes by the STRT method (Katayama et al., 2013; Töhönen et al., 2015). The method is an extension of SAMseq (Li and Tibshirani, 2013), a non-parametric approach for identifying differential expression in RNA-seq data.

To analyze the differential expression induced by the overexpressed gene, we compared the GFP-positive samples from each transfection with three sets of controls: GFP-positive samples from mCherry control, GFP-negative samples from both mCherry and the gene of interest, and GFP-negative samples from the whole library (each separately with q<0.1) (Fig. 3C). For robustness, the intersection of these three gene lists was used for further analyses. The number of upregulated and downregulated TFEs by their genomic locations is shown in Fig. 3D. More than 2500 TFEs were upregulated and 500 downregulated by LEUTX.n. The number of differentially expressed TFEs by either LEUTX.R or OTX2 suggested more modest effects with higher repressor than inducer activity (Fig. 3D). The overexpression of DPRX was followed by a massive downregulation of TFEs, suggesting that it acts as a repressor rather than an inducer. In all comparisons, most of the TFEs mapped to the 5′UTR of coding exons of coding genes. However, the results also showed a number of TFEs without previous annotation, suggesting novel transcripts from previously unannotated TSSs. These novel TSSs might be developmental stage specific and/or encode novel transcript variants. Because of the lack of functional information for novel TFEs, we focused further analyses on the TFEs located at the 5′UTR of the annotated, coding genes. The TFEs, their corresponding genes and genomic annotations are given in Table S2. The top 20 genes upregulated by LEUTX.n are shown in Table 1. The expression of the experimental target genes of LEUTX.n in human preimplantation embryos was confirmed using the Yan et al. (2013) dataset (Fig. S9). The LEUTX.n targets included the pluripotency-associated genes *DPPA3* (also called *STELLA*), *HESX1* and *KAT7*, whereas the LEUTX.R isoform had no effect on these genes (Table S2). We then applied the GOrilla tool (Eden et al., 2009) to analyze Gene Ontology (GO) category enrichment among the genes upregulated by LEUTX.n. All three categories – biological processes, molecular function and cellular component – revealed enrichment related to fundamental functions of the cell: RNA binding, protein localization, translation, gene expression and ribosomal unit (Table 2, Fig. S10).

**Table 2.**
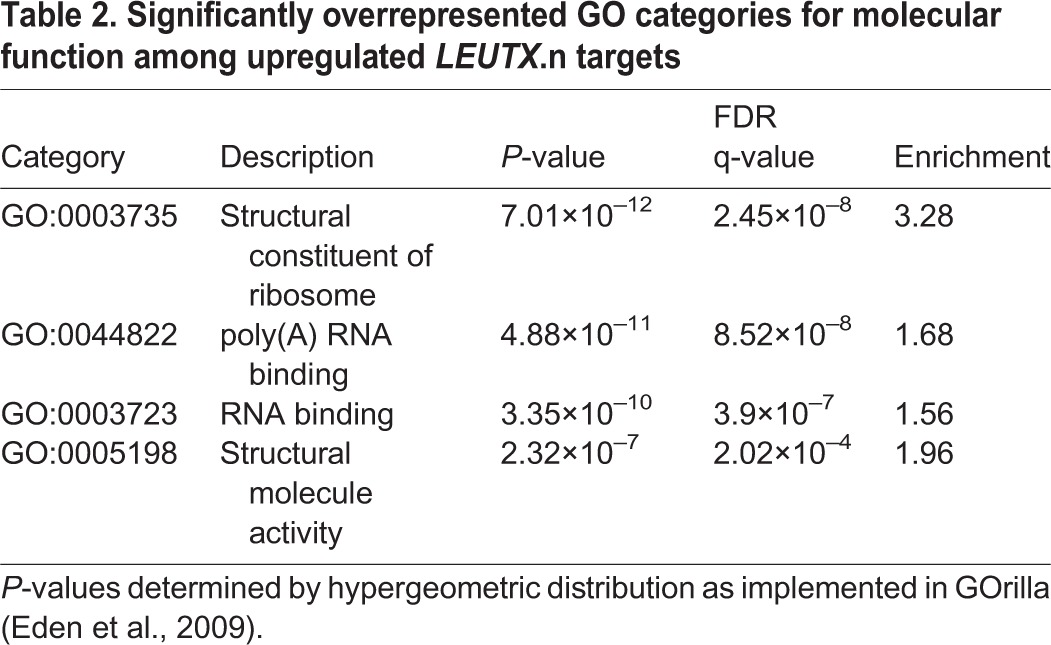
**Significantly overrepresented GO categories for molecular function among upregulated *LEUTX*.n targets**

### *LEUTX*.n and *DPRX* affect genes that are activated in early embryos

**Fig. 4.**
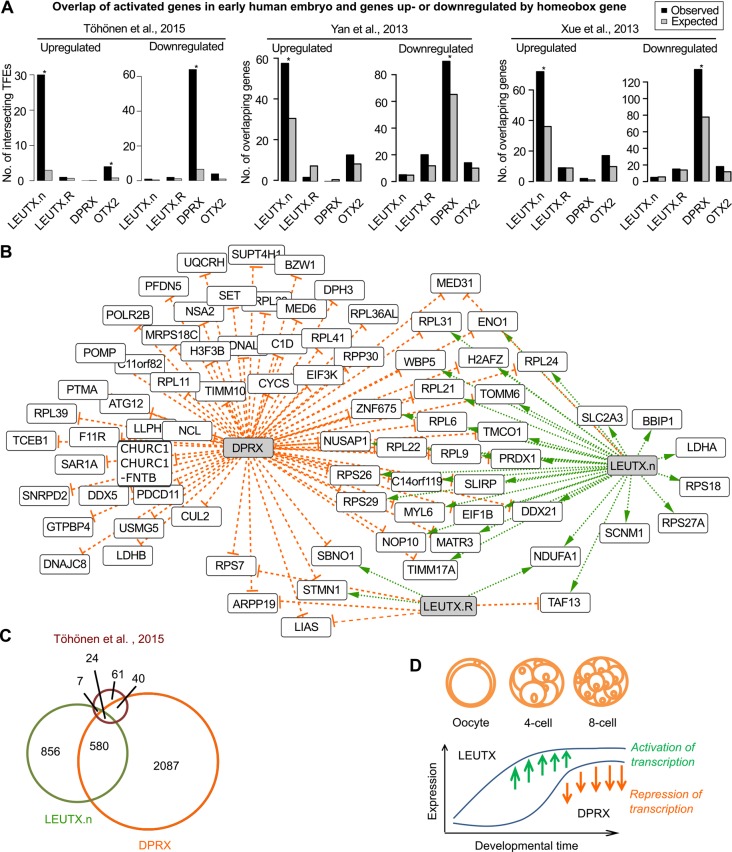
***LEUTX.*n upregulates genes that are activated in the early embryo.** (A) Overlap of upregulated or downregulated genes and activated genes in the early embryo was evaluated by Chi-squared test (FDR=0.05). The observed and expected number of overlapping genes was calculated for three independent datasets: Töhönen et al. (2015), Yan et al. (2013) and Xue et al. (2013). The RPKM values for upregulated genes at either the 4- or 8-cell stage (FDR<0.05) were calculated as described in the supplementary Materials and Methods. (B) Gene network showing the overlapping genes between Töhönen et al. (2015) and targets of *LEUTX* or *DPRX*. Orange T-arrows represent downregulation and green arrows indicate upregulation. (C) The number of overlapping targets of *LEUTX*.n and *DPRX* (604 observed/257 expected) and the genes activated in early embryos (24 observed/2 expected) (Töhönen et al., 2015) visualized as a size-proportional Venn diagram. *P*<1.0×10^–6^ for each (binomial distribution). (D) Summary of the *LEUTX.*n and *DPRX* expression patterns in human preimplantation embryos (Töhönen et al., 2015).

The detection of *LEUTX*.n in human preimplantation embryos but not in adult tissues suggested its importance during early development. *DPRX*, on the other hand, was upregulated at the 8-cell stage, thus being a potential target gene of LEUTX, but it acted as a potent repressor of transcription in the overexpression experiment in hESCs. We compared the experimental overexpression targets with the early activated genes from three independent datasets (Yan et al., 2013; Xue et al., 2013; Töhönen et al., 2015). In all three datasets, the genes activated in 4-cell to 8-cell stage embryos were highly enriched among the experimentally upregulated targets of LEUTX.n (FDR<0.05, Chi-squared test), whereas the targets of LEUTX.R showed no significant difference in the observed and expected numbers (Fig. 4A). Furthermore, the downregulated targets of DPRX showed a larger than expected overlap among the embryo activated genes.

The overlap between the target genes for LEUTX.n, LEUTX.R and DPRX and the early activated genes described by Töhönen et al. (2015) is visualized as a gene network (Fig. 4B). Furthermore, the number of overlapping genes between LEUTX.n and DPRX targets and early activated embryonic genes described by Töhönen et al. (2015) is shown as an area-proportional Venn diagram (Fig. 4C). We observed remarkable overlaps between LEUTX.n and DPRX targets (604 genes observed, 257 expected by chance) and between genes that were regulated both by LEUTX.n and DPRX and also activated in early embryos (24 genes observed, 2 expected; *P*<1.0×10^–6^ for both comparisons, binomial distribution).

We hypothesize that LEUTX.n might regulate the EGA by inducing the first transcripts in the human embryo, and that DPRX, activated one cell division later, would repress the expression of the same genes. The expression patterns of *LEUTX*.n and *DPRX* at oocyte, 4-cell and 8-cell stage are summarized in Fig. 4D (data from Töhönen et al., 2015).

### LEUTX and DPRX both target the same DNA motif

**Fig. 5.**
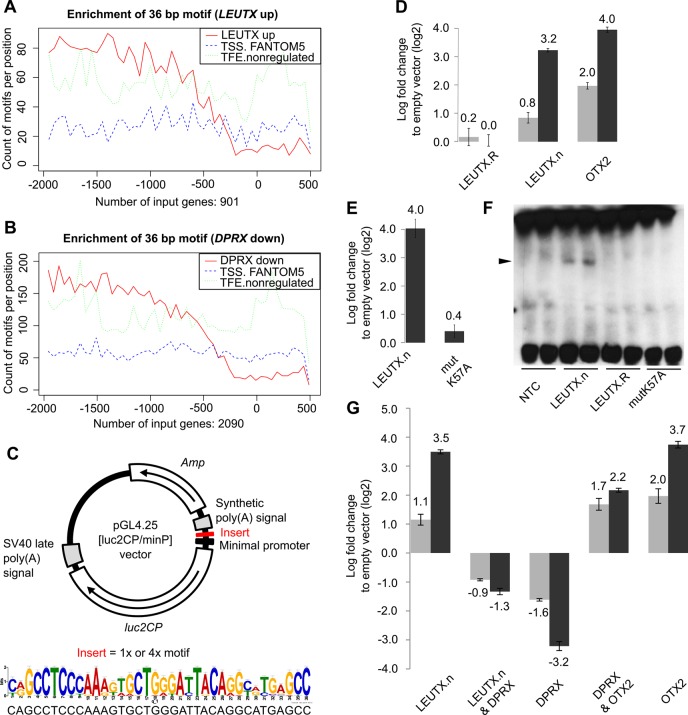
***LEUTX*.n and *DPRX* act on a motif predicted to play a central role in early human embryos.** (A,B) MAST analysis for (A) genes upregulated by *LEUTX.*n (*n*=901) and (B) downregulated by *DPRX* (*n*=2090) using the consensus motif visualized in C. The solid red lines show the count of motifs in *LEUTX* and *DPRX* target genes, while blue and green dotted lines shows counts on equal numbers of start sites from FANTOM5 database and non-regulated TFEs, respectively. (C) The luciferase vector pGL4.25 containing a minimal promoter and the 36 bp motif. (D,E) Log_2_ fold changes in luciferase expression between motif-containing and corresponding empty vector with cotransfection of *LEUTX*.n, *LEUTX*.R or *OTX2* (D) and with cotransfection of non-mutated or mutated (mutK57A) *LEUTX*.n (E). (F) EMSA showing that LEUTX.n, but not LEUTX.R or mutated LEUTX.n, binds to the 36 bp motif (*n*=3). Samples are shown in technical duplicates. NTC, non-template control refers to reticulocyte lysate only. (G) Log_2_ fold changes in luciferase expression between cotransfection of *LEUTX.*n, *DPRX* or *OTX2* alone or in combination. In D,E,G, gray and black columns represent 1 and 4 repeats of the motif, respectively. Experiments were performed in three biological replicates with two technical replicates each. Error bars indicate mean±s.e.m.

A *de novo* predicted 36 bp DNA motif was found enriched among the promoters of early activated genes and suggested to play a role in EGA (Töhönen et al., 2015). In order to test whether this motif is enriched in the promoter region of experimentally regulated genes, we applied the motif enrichment analysis (MAST) (Bailey and Gribskov, 1998) to the *LEUTX*.n and *DPRX* target gene promoters at genomic positions −2000 to +500 bp around the regulated TFEs. An equal number of non-regulated TFEs or random start sites from FANTOM5 were used as control datasets. The analysis showed that the 36 bp motif was indeed enriched among the promoter regions of the experimentally regulated genes (Fig. 5A,B).

To experimentally confirm that LEUTX and DPRX can mediate their effects through the 36 bp motif sequence, we constructed luciferase reporter vectors with one copy or four tandem copies of the 36 bp sequence (Fig. 5C), cotransfected these with the PRD-like homeobox genes into HEK-293 cells, and measured the luciferase signals 24 h after transfection. We first studied the ability of LEUTX.n and LEUTX.R isoforms to act on the motif and induce luciferase expression. The results show that cotransfection with the *LEUTX*.n isoform induced the luciferase signal up to 10-fold, whereas *LEUTX*.R did not have any effect (Fig. 5D), further supporting LEUTX.n as the functional LEUTX isoform. We transfected *OTX2* as a reference gene with a previously shown binding site (GGATTA) (Briata et al., 1999; Hoch et al., 2015), which is also part of the 36 bp motif, and it showed up to 15-fold induction of luciferase expression. In order to study whether LEUTX.R can act as dominant-negative isoform, we performed a similar luciferase assay by cotransfecting *LEUTX*.R with *LEUTX*.n or *OTX2*. Cotransfection only mildly attenuated the luciferase signals, thus suggesting that LEUTX.R does not act as a dominant negative (Fig. S11). We then studied whether LEUTX.n binds directly to the motif by mutating the *LEUTX*.n expression construct at a single amino acid at a central position of the homeodomain (K57A), similar to previous studies on *bicoid* in *Drosophila melanogaster* (Hanes and Brent, 1989). The mutation abolished LEUTX.n function in the luciferase assay (Fig. 5E). Furthermore, electrophoretic mobility shift assay (EMSA) revealed binding of LEUTX.n, but not LEUTX.R or mutated LEUTX.n, to the 36 bp motif (Fig. 5F, Fig. S12).

Interestingly, also the DPRX binding sites (NRGATTAKCYN and NNRGATTADN) predicted using systematic evolution of ligands by exponential enrichment (SELEX) overlap with the 36 bp motif (Jolma et al., 2013). We then performed the luciferase reporter assay to study the effect of *LEUTX*.n, *DPRX* and *OTX2* alone and in combination. The data showed that both *LEUTX*.n and *OTX2* induced luciferase expression compared with the empty vector, whereas *DPRX* suppressed the expression (Fig. 5G). *DPRX* also reduced luciferase expression when cotransfected with either *LEUTX.*n or *OTX2*. Similar results as those in 24 h transfections were obtained at the 10 h time point, suggesting that both LEUTX.n and DPRX act directly on the 36 bp motif (Fig. S13). This finding suggested that DPRX might be a direct competitor for the DNA binding motif and that it might counteract the effect of LEUTX through binding to the same 36 bp motif.

## DISCUSSION

Human EGA is poorly understood. Knowledge concerning what governs differentiation and pluripotency might be highly useful for stem cell research and regenerative medicine. In our recent study, we used improved single-cell promoter-tagging RNA sequencing methods and transcript quantification on external normalization rather than the commonly used relative proportion methods. We previously identified putative new transcription factor genes and implicated the PRD-like homeobox genes as key players in human EGA (Töhönen et al., 2015). Here, we describe the molecular cloning and functional characterization of the novel *LEUTX* variant that encodes a complete homeodomain (*LEUTX*.n) as well as the earlier RefSeq annotated isoform with an incomplete homeodomain (*LEUTX*.R). Comparison of the target gene profile of LEUTX.n and genes upregulated in 4-cell and 8-cell embryos suggested a key role for LEUTX.n in EGA.

*LEUTX* is one of the first genes expressed in human preimplantation embryos. Here, we confirmed *LEUTX* expression in human 8-cell blastomeres on the RNA and protein level and, in addition, we show the relatively low-level expression of *LEUTX* in hESCs. Our data, as well as previously published datasets, support the expression of *LEUTX* in pluripotent cells but not in any terminally differentiated cells (Forrest et al., 2014; Lizio et al., 2015). An exception is the *LEUTX*.n expression implicated in FSHD, suggesting that dysregulated *DUX4* might induce *LEUTX*.n expression in diseased muscles, which might disturb cell differentiation (Yao et al., 2014). These findings suggest that *LEUTX* expression is restricted to human preimplantation development and that *LEUTX* might play a key role in EGA.

Further support was gained by the profiling of target genes in hESCs overexpressing *LEUTX*.n. The gene targets included known pluripotency genes, such as *DPPA3* and *HESX1*. *Dppa3* (also known as *Stella* or *Pgc7*) was initially observed in mouse primordial germ cells (Sato et al., 2002), characterized as a maternal effect gene in mouse (Payer et al., 2003), and suggested to be a marker for pluripotency in both mouse and human (Bowles et al., 2003). Furthermore, *Dppa3* has been shown to be a direct downstream target of *Tbx3* (Waghray et al., 2015) that is necessary for the maintenance of pluripotency. Although the importance of *Dppa3* has been shown, little is known about *Dppa3* regulation. *Hesx1* has been characterized as encoding a PRD-like homeodomain factor that acts as a repressor and is connected with pituitary organogenesis (Dasen et al., 2001). Furthermore, it is expressed in murine ESCs and has been shown to be essential in maintaining pluripotency (Li et al., 2015; Webb et al., 1993). Interestingly, *NANOG* is also among the upregulated targets of *LEUTX*.n.

Direct evidence for the binding and functionality of LEUTX.n but not LEUTX.R or mutated LEUTX.n came from EMSA and luciferase reporter assays using the previously predicted 36 bp EGA-specific DNA binding motif (Töhönen et al., 2015). Further experiments suggested that both LEUTX.n and OTX2 can induce expression through the 36 bp EGA motif, and that DPRX acts as a partial repressor. The computationally tested specificity of LEUTX.n and DPRX binding to target gene promoters indeed indicated that the predicted motif is significantly enriched at positions −2000 to +500 bp from the TSSs. The novel finding of DPRX as a repressor of embryo activated genes may suggest a further regulatory mechanism in EGA involving the selective repression of activated genes after the 8-cell stage.

Early embryo development is still largely uncharacterized in humans. However, in model systems such as *Drosophila*, a number of key observations have been made that might also have relevance to human EGA. For example, similar to the 36 bp motif in human, the so-called TAGteam motif identified in *Drosophila* has been shown to be enriched in the upstream regions of genes activated around the time of the maternal-to-zygote transition (ten Bosch et al., 2006). Furthermore, key transcription factors, such as Zelda and Stat92E, have been shown to act on the TAGteam motif and control transcription of the zygotic genome at the very beginning of embryonic development (Tsurumi et al., 2011; Liang et al., 2008). Interestingly, the 36 bp regulatory motif was identified to frequently overlap with Alu elements (Töhönen et al., 2015), which are primate-specific retrotransposon elements. In mouse, EGA is controlled via murine-specific long terminal repeat (LTR) retrotransposons and murine-specific endogenous retroviruses (Macfarlan et al., 2012; Peaston et al., 2004). However, at EGA in mouse, the murine-specific retroelements were shown to be frequently transcribed as an alternative 5′ exon of host genes, whereas the 36 bp motif in human EGA is usually located far upstream of the TSSs. Although the *LEUTX* promoter itself contains a similar sequence to the 36 bp motif, the human *LEUTX* TSS does not overlap with retroelements. Therefore, it seems that the regulation of not only *LEUTX*, but also EGA, is evolutionarily optimized for each mammalian species.

In conclusion, we propose that the complete homeodomain LEUTX.n isoform is the functional LEUTX isoform that is suggested to play a key role in early embryo development and pluripotency. LEUTX activates the first genes in human preimplantation development by acting on the predicted DNA sequence motif, and some of the early activated genes are then later repressed by DPRX.

## MATERIALS AND METHODS

### Human preimplantation embryos, hESCs and cDNA generation

The sample sources and methods for sample preparation have been described (Töhönen et al., 2015). Human preimplantation embryos used for this study were donated by couples who underwent infertility treatment by *in vitro* fertilization (IVF), and they were collected in Sweden (ethics approvals Dnr 2010/937-31/4 and 2012/1765-31/1 of the Regional Ethics Board in Stockholm). Cryopreserved cells not needed for IVF and destined for destruction since the legal storage time had been reached were donated by informed consent. None of the donors received any financial reimbursement. hESC lines HS401, HS980, HS983a and H9 were obtained from the laboratory of O.H.

For cDNA library preparation, human 4-cell stage embryos were thawed (ThawKit™ Cleave, Vitrolife) and cultured in G-1™ Plus medium (Vitrolife) overnight under standard conditions as performed in the IVF Clinic (5% CO_2_/5% O_2_), allowing them to develop until 8-cell stage. Each embryo was then collected into a 0.5 ml PCR tube containing 4.45 µl fresh lysis buffer prepared according to published protocols (Tang et al., 2010). In total, three single 8-cell libraries were prepared.

### STRT RNA-seq of human preimplantation embryos

The data on human preimplantation embryos and data processing using the STRT RNA-seq method were previously described by Töhönen et al. (2015). The processed STRT reads supporting the results of the present study are available in the European Nucleotide Archive (http://www.ebi.ac.uk/ena/data/view/PRJEB899). TFE for *LEUTX* is FE270433 (chr19:40269482-40269570, +). The normalized expression values used for drawing beeswarm plots are given in Table S3.

### Quantitative PCR (qPCR)

Total RNA extracted from three hESC lines (H9, HS401 and HS980) in three biological replicates of each was converted to cDNA using SuperScript III First-Strand Synthesis SuperMix for qRT-PCR (Invitrogen, 11752) according to the manufacturer's instructions. qPCR assay was performed using 22 ng cDNA from each cell line sample and 10 ng human 8-cell library that was used for cloning of the genes. qPCR was carried out using an ABI PRISM 7500 Fast Real-Time PCR System with FastStart Universal SYBR Green Master Mix (Roche) according to the manufacturer's instructions. The primer sequences are given in Table S4. To confirm the qPCR amplicon, it was cloned into pCRII-dual promoter TOPO vector using the TOPO TA cloning kit (Invitrogen), and the sequence was verified by Sanger sequencing (Eurofins Genomics).

### Construction of expression vectors

In order to overexpress *LEUTX.*R (NM_001143832.1), *LEUTX.*n, *DPRX* and *OTX2* (NM_172337) in human cells, the ORFs were cloned into a modified pFastBac expression vector CMVe.EF1α.eGFP-WPRE that was kindly provided by Prof. Shu Wang (Institute of Bioengineering and Nanotechnology, Singapore) (Du et al., 2010). The modifications are described in detail in the supplementary Materials and Methods and by Töhönen et al. (2015).

ORFs for *LEUTX* and *DPRX* were amplified from the TOPO vectors containing the full-length clone (European Nucleotide Archive accession numbers LN651090 and LN651088, respectively) and *OTX2* was amplified from a mixture containing equal amounts of cDNAs originating from human placenta, testis and hESC lines HS401 and H9. The ORF for predicted *LEUTX.*R was amplified from the same TOPO vector as the novel isoform, but using a forward primer 90 bp downstream of novel start site (Fig. 1A). The amplification primers included *Asc*I and *Pac*I restriction sites (primer sequences are given in Table S5). The PCR products were digested using *Asc*I and *Pac*I (New England Biolabs) and ligated into pFastBac vector digested with the same enzymes. mCherry fluorescent protein was used as a control and amplified from the standard injection marker construct elt-2::GFP for *C.elegans* (a kind gift from Gert Jansen, The Erasmus University Medical Center, Rotterdam, The Netherlands). In order to mutate *LEUTX*.n in the pFastBac vector, the QuikChange II Site-Directed Mutagenesis Kit (Agilent) was used according to the manufacturer's instructions and the primers described in Table S6.

*LEUTX*.n and *LEUTX*.R were further cloned into the pcDNA3.1/V5-His-TOPO vector (Invitrogen) from the TOPO vector containing the full-length clone LN651090. The primer sequences, including either *Bam*HI or *Not*I restriction site at their 5′-end, are given in Table S7.

### Indirect immunofluorescence labeling

Four-cell stage embryos were thawed using ThawKit™ Cleave following the manufacturer's instructions and cultured overnight on G-1™ medium, allowing them to develop until the 8-cell stage. Embryos were fixed in 4% paraformaldehyde for 15 min, permeabilized in 0.3% Triton X-100 in PBS, and treated with anti-LEUTX antibody (NBP1-90890, Novus Biologicals; 1:100) overnight at 4°C followed by donkey anti-rabbit IgG Alexa Fluor 647 (A31573, Molecular Probes; 1:1000) for 2 h at room temperature and Hoechst 33342 nuclear stain (H3570, Molecular Probes; 1:1000) for 20 min at room temperature. Images were acquired with a Zeiss LSM710-NLO point scanning confocal microscope. Post-acquisition analysis was carried out using Imaris (Bitplane). Control staining to validate antibody specificity is described in the supplementary Materials and Methods.

### Western blot

Total protein extracts from HEK-293 cells overexpressing *LEUTX*.n or *LEUTX*.R were analyzed by western blot as described in the supplementary Materials and Methods.

### Overexpression of *LEUTX* in hESCs and FACS for STRT RNA-seq

HS401 cells were cultured on Laminin-521 (Biolamina) in mTeSR™1 medium (STEMCELL Technologies). Fully confluent cells were trypsinized, washed with Dulbecco's phosphate buffered saline, and suspended in 50 µl transfection solution containing 1 µg pFastBac expression vector with 3 µl Lipofectamine 2000 (Invitrogen) in DMEM. The modified pFastBac overexpression vector contains the gene of interest, an IRES element and eGFP for simultaneous expression of the target gene and a fluorescent marker in the same cells. The cell suspension was transferred to a fresh Laminin-521-coated well, the cells were allowed to settle in the transfection solution for 15 min, and the medium was subsequently changed to mTeSR™1.

FACS was performed on trypsin-treated cells 9-11 h after transfection. Cells were sorted by BD FACSAria III using an 85 µm nozzle according to their eGFP expression to positive or negative wells. Clustered cells and debris were excluded by FSC and SSC gates, and dead cells were excluded by propidium iodide viability stain. GFP-positive cells were sorted in triplicate (3×75 cells for novel *LEUTX*, *DPRX* and *OTX2*) or quadruplicate (4×50 cells for RefSeq *LEUTX*) into 5 µl STRT lysis buffer and two STRT libraries were prepared. Equal numbers of GFP-negative cells per transfection were sorted in triplicate.

### STRT RNA-seq library preparation and data analysis

STRT sequencing libraries were prepared for two purposes: (1) single-cell expression profiling in two hESC lines and single 8-cell blastomeres and (2) target gene detection following overexpression experiments. The first library included 16 human single blastomeres from 8-cell stage embryos, 16 human regular hESCs (line HS980), 16 human single 8-cell blastomere-derived hESCs (line HS983a), all picked manually under a microscope into 5 µl lysis/cDNA synthesis buffer. Thawing of human embryos for STRT is described in the supplementary Materials and Methods.

STRT sequencing libraries consisting of 48 samples were prepared for sequencing on an Illumina HiSeq 2000 platform according to a modified STRT protocol (Krjutškov et al., 2016). STRT sequencing data analysis is described in the supplementary Materials and Methods and Table S9. Pre-processing of STRT reads and alignments were performed using an established analysis pipeline (Krjutškov et al., 2016). The data normalization and differential expression analyses were performed using the R package SAMstrt (Katayama et al., 2013).

STRT reads supporting the results of the present study are available in the European Nucleotide Archive (http://www.ebi.ac.uk/ena/data/view/PRJEB12467 and http://www.ebi.ac.uk/ena/data/view/PRJEB12453). The normalized reads and TFE locations for the overexpression experiment can be visualized as a ‘my hub' on the UCSC genome browser (http://jke.biosci.ki.se/hubs/Lib345/hub.leutx.txt).

### Construction of luciferase reporter vector and the luciferase assay

In order to study the recently discovered DNA motif – referred to as the 36 bp *de novo* motif (Töhönen et al., 2015) – a 216 bp synthetic construct containing four motifs in tandem with intervening restriction sites was constructed (Eurofins). The construct sequence is given in the supplementary Materials and Methods. The construct containing the 4×36 bp *de novo* motif was restricted with *Sfi*I and ligated into restriction digested pGL4.25 [luc2CP/minP] luciferase reporter vector (Promega). The pGL4.25 luciferase reporter vector contains a minimal promoter. To obtain a construct with only one repeat of the 36 bp *de novo* motif, the construct in pGL4.25 was subjected to three cycles of digestion, purification and religation using *Eco*RV, *Nhe*I and *Sac*I (all from New England Biolabs) in that order. Construct sequences were verified by Sanger sequencing (Eurofins Genomics).

The luciferase reporter assays were performed as described by Töhönen et al. (2015).

### EMSA

EMSA was performed using proteins synthesized with the TNT T7 Quick for PCR DNA (Promega) transcription/translation system according to the manufacturer's protocol. Primer sequences are given in Table S8. For details, see the supplementary Materials and Methods.
